# Enhanced Extraction of Activation Time and Contractility From Myocardial Strain Data Using Parameter Space Features and Computational Simulations

**DOI:** 10.1155/2024/1059164

**Published:** 2024-10-12

**Authors:** Borut Kirn

**Affiliations:** Medical Faculty, University of Ljubljana, Ljubljana, Slovenia

**Keywords:** activation time, computational modeling, contractility, myocardial strain, proximity map

## Abstract

A computational model enables the extraction of two critical myocardial tissue properties: activation time (AT) and contractility (Con) from recorded cardiac strains. However, interference between these parameters reduces the precision and accuracy of the extraction process. This study investigates whether leveraging features in the parameter space can enhance parameter extraction. We utilized a computational model to simulate sarcomere mechanics, creating a parameter space grid of 41 × 41 AT and Con pairs. Each pair generated a simulated strain pattern, and by scanning the grid, we identified cohorts of similar strain patterns for each simulation. These cohorts were represented as binary images—synthetic fingerprints—where the position and shape of each blob indicated extraction uniqueness. We also generated a measurement fingerprint for a strain pattern from a patient with left bundle branch block and compared it to the synthetic fingerprints to calculate a proximity map based on their similarity. This approach allowed us to extract AT and Con using both the measurement fingerprint and the proximity map, corresponding to simple optimization and enhanced parameter extraction methods, respectively. Each synthetic fingerprint consisted of a single connected blob whose size and shape varied characteristically within the parameter space. The AT values extracted from the measurement fingerprint and the proximity map ranged from −59 to 19 ms and from −16 to 14 ms, respectively, while Con values ranged from 48% to 110% and from 85% to 110%, respectively. This study demonstrates that similarity in simulations leads to an asymmetric distribution of parameter values in the parameter space. By using a proximity map, this distortion is considered, significantly improving the accuracy of parameter extraction.

## 1. Introduction

Speckle-tracking echocardiography (STE) allows for the noninvasive assessment of ventricular wall mechanics by revealing changes in myocardial strain patterns [1, 2]. Two key pathological subgroups that alter cardiac mechanics include those affecting action potential conduction, leading to varied contractions across myocardial regions, and those reducing myocardial contractility (Con). Examples of these conditions are left bundle branch block (LBBB) [3] and myocardial infarction [4].

Given the widespread accessibility of STE, it provides significant benefits in diagnosing and monitoring cardiac conditions [5, 6]. This utility would be further enhanced by mapping myocardial properties such as activation time (AT) and Con from strain patterns. Mapping AT and Con could aid in selecting patients for cardiac resynchronization therapy (CRT) [7] and evaluating novel heart failure treatments [8]. However, mapping AT and Con poses substantial challenges.

Previous studies have attempted to derive surrogate values for AT and Con by identifying prominent features in the strain pattern, such as the onset of shortening during systole, the time to the first peak of shortening, and strain at the end of systole [9]. These methods, however, are prone to nonlinearity and may not accurately capture subtle changes in tissue properties.

To overcome these challenges, a model-based approach has been proposed that integrates detailed information on regional variations in tissue characteristics, allowing the model to accommodate nuanced alterations in tissue properties across different regions of the heart [10–13]. Solving this inverse problem—employing computational models to estimate parameter values by, for example, using a simple optimization method to find the best fit between simulated and measured values—may encounter difficulties in ensuring uniqueness and accuracy in parameter extraction, as different parameter sets could equally describe the observed data [14–16].

In the parameter space, this nonuniqueness of the solution manifests as regions of model parameter sets that yield similar results. These regions may be fragmented or connected, forming unevenly shaped blobs, with shapes varying in different regions of the parameter space. Thus, the image of a measured signal in the parameter space may be highly specific and characteristic for the signal. The question is whether these distributions of inverse problem solutions in the parameter space could, instead of being a drawback, be utilized in solving the inverse problem itself.

The objective of this study is to develop a technique to use the characteristic shapes of measurement images in the parameter space to solve the inverse problem and to assess the theoretical limitations of parameter extraction uniqueness when using this approach. The study is using a Hill-based computational model of sarcomere mechanics to simulate strain patterns for various AT and Con and the strain data from LBBB patients, as they pose a unique challenge for analysis during systole due to complex interactions between early and late activated myocardial regions.

## 2. Methods

### 2.1. Computational Model

**Figure 1:**
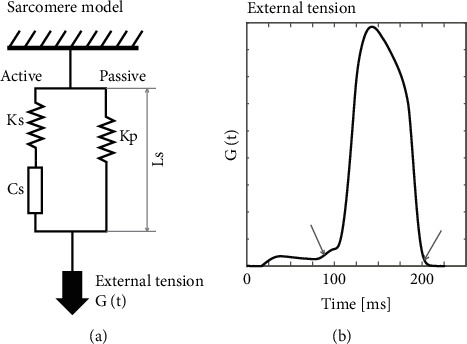
(a) Schematic presentation of a modified three-element Hill-based model of sarcomere mechanics. The “Active” parallel branch is composed of a passive elastic element (Ks) and a contractile element (Cs), and the “Passive” branch has a passive elastic (Kp) element. The length of the sarcomere element (Ls) is used to calculate strain as a function of time given that external tension G(t) and tension generated by the contractile element Cs are both a function of time. (b) The time pattern of external tension G(t); the arrows mark closure and opening of the mitral valve.

A lumped parameter computational model of sarcomere mechanics was employed to simulate strain in the left ventricular wall, producing synthetic measurements. The model used was a modified three-element Hill-based model consisting of three main components: contractile element (Cs), an elastic element (Ks) in series with Cs, and another elastic element (Kp) in parallel with Cs and Ks as shown in Figure 1(a). The Cs component represents the muscle fibers responsible for generating force during contraction whereas Ks and Kp represent the passive stiffness of sarcomere and surrounding tissue, respectively. The external tension *G*(*t*) exerted on the sarcomere element was kept constant throughout the simulated cardiac cycle regardless of the mechanics of the simulated sarcomere. This tension, derived from a heart failure patient simulation, drives sarcomere contraction. The time pattern of external tension (Figure 1(b)) was obtained from a heart failure patient simulation of a CircAdapt closed-loop cardiovascular model.

The rationale behind the constant external tension *G*(*t*) is that the simulated sarcomere was a very small part of the ventricular wall mass (0.01%) and thus its influence on the overall mechanics of the ventricle was negligible. In this way, a situation was mimicked in which the gross mechanics of the ventricle remain the same despite changing local tissue parameters (AT and Con in the small sarcomere), which made it possible to study the isolated influence of AT and Con on the mechanics of the local tissue. The gross ventricular mechanics may in general be composed of locally variable tissue properties, which interaction sums up into one shared external tension that is equal for all local parts in the ventricular wall [17, 18].

In the model, the stiffness of the contractile element was a phenomenological representation of the density of cross-bridge formation, and its time derivative was modeled as a function of sarcomere length (*L*_si_) and time *t*, accounting for the experimentally observed length dependence of activation and force-velocity relation of cardiac myofibers [19, 20]. A full description of the sarcomere model was previously published by Walmsley et al. [18].

From the sarcomere length *L*_*s*_, a myofiber strain *E*_*f*_ was calculated as

(1) $$\begin{array}{c} E_{f}\left(t\right)=\frac{L_{s}\left(t\right)}{L_{s,0}}-1, \end{array}$$

where *L*_*s*,0_ denotes the sarcomere length at the time of closure of the mitral valve. The temporal resolution of the simulation was 2 ms.

**Figure 2:**
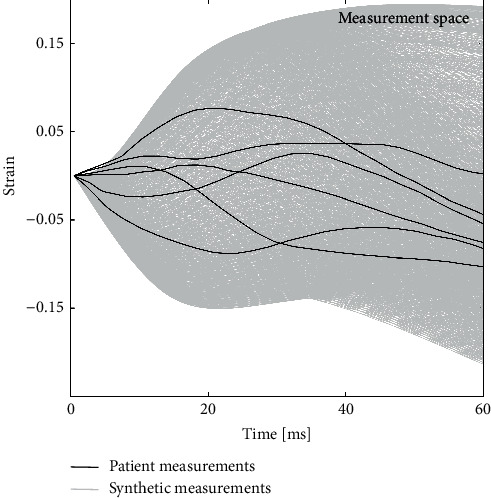
Patient strain data (black): six patterns of a patient with left bundle branch block measured with speckle-tracking echocardiography (four-chamber view, longitudinal strain) (Risum et al.). Synthetic measurements (background gray): 1,681 simulated strain patterns, each with a different set of time of activation (AT) and contractility (Con) parameters.

Altogether, 1681 simulations were conducted, each with a different set of AT and Con values (Figure 2). In the simulation, AT, representing the time difference between the activation of the contractile element and the onset of external force, was varied across a spectrum from −100 to 100 ms. This range was determined based on the QRS duration of patients with LBBB, encompassing both delayed and advanced activation scenarios relative to the onset of external force, which could reflect physiological conditions such as conduction delays or early activation patterns observed in cardiac pathologies. The Con parameter in the simulation was a scaling factor of contractile element maximum tension. Ranging from 2% to 202%, with 5% increments, this parameter allows us to explore a wide range of contractile strengths. At 2%, it represents the total loss of Cont while at 202%, it indicates peak stimulation of muscle. A value of 100% represents the baseline contractile strength, while values above or below 100% signify increased or decreased contractility, respectively. This range enables the investigation of both hypercontractile and hypodynamic states, which are pertinent to various cardiac conditions.

Specifically, when AT was set to 0 ms and Con to 100%, the contractile element tension development pattern in the sarcomere perfectly mirrored the pattern used in generating external tension. This configuration serves as a reference point for comparison and ensures consistency in the simulation setup.

### 2.2. Patient Data

This study used the data of a patient with LBBB. LBBB pathology was selected because its pathophysiological origin is blocked electrical signal conduction path which mostly influences myocardial AT and should have little if any influence on its Con. Thus, the extraction of AT and Con should reflect it accordingly. The measurements were previously published by Risum et al. [21]. They used STE (four-chamber view) to measure six longitudinal strain patterns distributed evenly around the left ventricular circumference (Figure 2). In the continuation of the manuscript, they are referred to as LBBB data strain patterns.

### 2.3. Inverse Problem Solution

**Figure 3:**
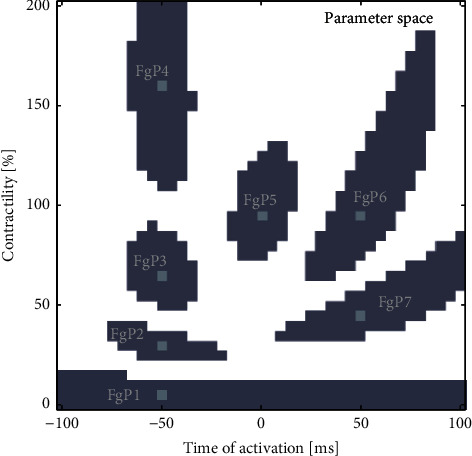
A selection of seven (FgP1–7) characteristically shaped synthetic fingerprints. Note: Fingerprints are superpositioned, and thus, each fingerprint is one gray blob with the rest of the square being white; the shape of each blob is different; the square within the blob marks the value of the simulation parameters.

The approach to solving the inverse problem was based on graphical comparisons of regions within the parameter space. To delineate these regions, the similarity of the signals in the measurement space was utilized. The measurement space consisted of strain patterns, which were either derived from patient data or generated through synthetic measurements (Figure 2). In contrast, the parameter space was structured as a grid of 41 × 41 fields, each representing pairs of AT and Con values. The region in the parameter space based on the similarity of strain patterns in the measurement space is referred to as a fingerprint.  Figure 3 presents selected synthetic fingerprints, labeled as FgP1 through FgP7. These fingerprints visually represent simulated strain patterns produced by the computational model, essentially creating an image in the parameter space with selected fields marked as binary.

The synthetic fingerprint (**FgP**_**Sy**(**TA**, **Con**)_) for each simulated strain pattern is created by identifying a cohort of similar strain patterns from the rest of the simulations. These cohort members are marked as binary in the parameter space, forming blobs of varying shapes (Figure 3). Each fingerprint is represented as a 41 × 41 binary matrix, resulting in a total of 1681 synthetic fingerprints, one for each location in the parameter space.  Figure 3 shows a selection of these synthetic fingerprints superimposed, providing insights into the diversity within the parameter space.

**Figure 4:**
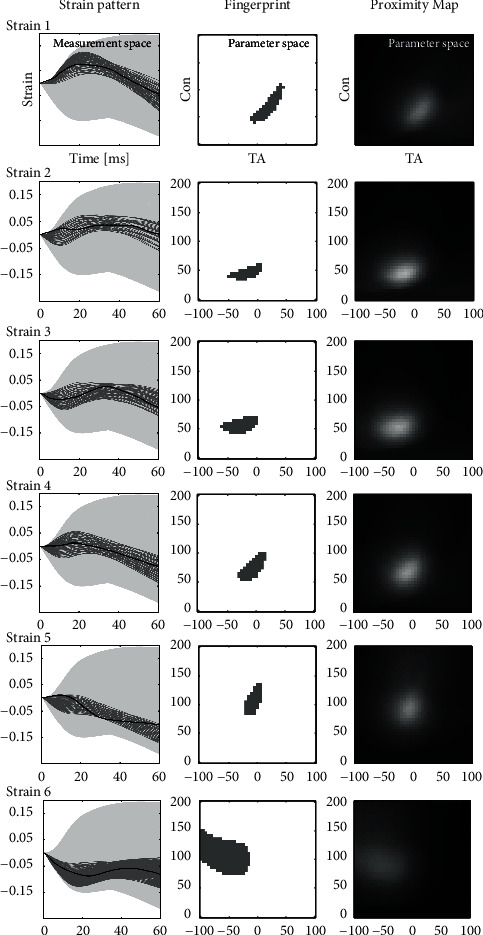
Six (1–6) measured strain patterns are shown in the colon strain pattern (black) accompanied by their cohort of similar synthetic strains (dark gray) and all simulated strain patterns (light gray background). The data fingerprint is shown in the colon fingerprint. Note that the fingerprint represents the cohort but now in the parameter space; the fingerprint is composed of one connected blob that shares characteristics in shape previously observed in synthetic fingerprints. In the proximity map, the brighter regions signify a high similarity between the data fingerprint and synthetic fingerprints.

Similarly, the data fingerprint (**FgP**_**Dj**_) is created for each data strain pattern by finding a cohort of similar strain patterns from all 1681 simulated patterns (Figure 4(a)). Strain patterns are considered similar if they differ by less than the realistic level of strain measurement error. The similarity (**S**_**p**,**i**_) of each strain pattern (*i*) to the reference strain pattern (*p*) is calculated as

(2) $$\begin{array}{c} S_{p,i}=\sqrt{\frac{\sum_{t=1}^{N}{\left(E_{p,t}-E_{i,t}\right)}^{2}}{N},} \end{array}$$

where *E* stands for fiber strain (equation (1)) and *N* runs from mitral valve closure until two-thirds into the duration of systole, which ends with mitral valve opening. For the strain patterns included in the cohort, the value of *S*_*p*,*i*_ was ≤ 0.03.

To summarize the alignment of features between the data and synthetic fingerprints, a proximity map was calculated. It is a representation of the reciprocal of the Euclidean distance between synthetic and data fingerprints. It is a 41 × 41 matrix where each element corresponds to the inverse of the mean Euclidean distance between the synthetic fingerprint and the data fingerprint. To compute this mean distance, the average distance between all nonzero points in the first matrix and all nonzero points in the second matrix is found.

When applied to a given data strain pattern, the resulting proximity map effectively summarizes the alignment of features between the data and synthetic fingerprints in terms of their position and shape. This method allows for a detailed comparison and enhances the accuracy of parameter extraction by leveraging the unique characteristics of the fingerprints in the parameter space.

### 2.4. Statistics

For each strain pattern data, a mean value and the standard deviation of AT and Con were calculated for the data fingerprint and proximity map images as a mass center of image intensity.

## 3. Results

Using a standard desktop PC computer, 1681 simulations were run within less than 3 min of computational time. The strain patterns obtained are shown in Figure 2.

The images of seven typical synthetic fingerprints are shown together in Figure 3. Each fingerprint is composed of one connected component (blob); however, each blob has a distinctively different shape. A visual review of all synthetic fingerprints confirmed these two characteristics. The shape in the region of higher contractility (Figure 3, FgP 4, 5, and 6) is more vertical, spreading across up to 100% in direction of the Con axes and 35 ms in the direction of the AT axes. In the region of low contractility, the blobs are stretched horizontally, spreading up to 100 ms in AT and 20% in Con (Figure 3, FgP 1, 2, and 7). In the regions of higher contractility and late activation, the shape of the blob is elongated in the direction of leaning (Figure 3, FgP 6 and 7). The asymmetrical shape of the blob reflects the nonlinearity of the parameter space. Although the criterion for strain pattern inclusion into the cohort was based on strain pattern proximity, equivalent to a normally distributed measurement error, the resulting distribution in the parameter space is not normal.

Each of the six LBBB strains was analyzed separately (Figure 4). For each, a cohort of similar simulated strain patterns is found (Figure 4, measurement space, dark gray). LBBB strains 1, 4, and 6 are positioned centrally within the cohort. In LBBB strains 2, 3, and 5, the position comes close to the cohort boundaries, suggesting a lesser degree of similarity between the measured and simulated strain patterns, particularly during early systole. Furthermore, the LBBB strains show intermediate shortening and stretching, the dynamics of which are lacking in simulated strains. The difference between simulated and measured strains highlights the insufficiency of the computational model in fully describing the physiological system. While the limitations in recreating the fine mechanics of the myocardial tissue are evident, the overall agreement with the measurements is also confirmed.

All LBBB fingerprints are composed of one connected component (Figure 4, fingerprint). The shape of the components shares some characteristics that were observed in the synthetic fingerprints, such as right leaning at high AT in measured strains 1 and 4 (Figure 4), and vertically elongated at high Con in measured strain 5 (Figure 4). The center of intensity in the proximity maps (Figure 4, proximity map) closely aligns with the position of blob in the fingerprint figure. Notably, the lower overall intensity in the proximity map of measured strain 6 signifies that the shape of the blob in its data fingerprint is very dissimilar to the shapes observed in the synthetic fingerprints.

Table 1 shows the results of the AT and Con mean values when extracted from the LBBB fingerprint image and proximity map. Measured strains 1 and 6 are late activated and early activated, as indicated by the starting positive and negative slope, respectively. The extracted AT values from both the fingerprint and proximity images are in accordance with this observation. However, there is an important difference between the two sets of results, which is reflected in the mean values of LBBB strains 2, 3, 4, and 5. LBBB strains 2, 4, and 5 are late activated, and measured strain 3 is early activated, as indicated by the starting slope. In the proximity map, the AT for these measured strains is more accurate than in the measurement fingerprint. In the proximity map, the AT for measured strains 2, 4, and 5 is −2, 2, and 2 ms, respectively, whereas in the LBBB fingerprint, they are −19, −6, and −7 ms, respectively. The range of AT and Con values is two to three times larger in the LBBB fingerprint results as compared to results from the proximity map. A large range in contractility is not expected in the LBBB patient, and thus the results from the proximity map seem more trustworthy with a range of 25%, compared to 65% in the results from the data fingerprint. The standard deviation in the data fingerprint varies significantly, whereas the results from the proximity map are very uniform although high.

## 4. Discussion

This study demonstrated that the similarity of simulations in the measurement space results in an asymmetric distribution of parameter values in the parameter space. By calculating a proximity map for parameter extraction, this distortion is considered, improving the accuracy of the process. The findings reveal that even when measurements are stochastically distributed in our observed physiological system, the extracted values are not. This highlights the importance of assessing the uniqueness of synthetic measurements, which introduces new information about the system and enhances the extraction of myocardial tissue properties (AT and Con) from measured local myocardial strain patterns in an LBBB patient.

The study's key contribution is that the LBBB fingerprint utilizes a stochastic concept of the distribution of underlying true values. The proximity map, on the other hand, applies the nonuniform distribution of underlying characteristics, as visualized in Figure 3. This approach better matches the complex and nonlinear reality of the system, providing a more accurate representation.

A conventional approach to parameter extraction involves comparing measured strain patterns to synthetic ones and identifying a cohort of similar signals from which the mean parameter values are calculated [10, 12]. In this study, this approach served as an intermediate step, as illustrated by the data fingerprint in Figure 4. By incorporating an additional step—creating a proximity map—new relevant information was introduced into the signal processing.

The shape of the blob in each synthetic fingerprint (Figure 3) is unique for each location within the parameter space. Each blob represents a cohort of strain patterns, and every location in the parameter space corresponds to a distinctive strain pattern. Thus, the shape of the blob reveals the internal characteristics of signal uniqueness.

Synthetic fingerprints uncover the internal characteristics of the system because the computational models they are based on are grounded in physiological findings and the laws of physics. Although these models are incomplete representations of complex biological systems, they embody several undeniable layers of causal relations reflected in the internal relationships between measurable values [14]. The proximity map, which is influenced by the asymmetrical distribution of parameter extraction uncertainty, adds further depth to this analysis—something that is not achieved by the simple optimization method. This asymmetry is significant, as evidenced by the comparison of FgP1 to FgP4 in Figure 3.

Applying the proximity map is an additional step toward aligning the measurements with the computational model. Therefore, the results should be interpreted within the context of the computational model used.

The improved extraction of AT and Con values is primarily based on analyzing the initial slope of the strain pattern and the range of AT and Con [9, 22]. According to basic sarcomere mechanics, the initial slope of the early-activated region is negative while that of the late-activated region is positive. However, the evidence for improved extraction accuracy remains indirect due to the lack of independently measured data on AT and Con along with the corresponding strain patterns. To the author's knowledge, no comprehensive published data currently includes measured strain patterns with independently verified AT and Con properties.

In the computational model, fiber strain was calculated, whereas in the measurements, left ventricular longitudinal strain was measured. Previous studies [23] have shown that these two measures are not directly correlated, with the primary difference lying in the magnitude of the strain rather than its pattern over time. This study relies on the relationships between signals, which are likely more conserved than the direct comparison of signal shapes. The estimated measurement error for myocardial strain measurement of ± 0.03 used in this study is based on the findings of Farsalinos et al. [24] and Donekal et al. [25]. Due to reported drift during ultrasound speckle-tracking measurements, the strain pattern analysis window was limited to the initial two-thirds of systole.

Future studies should evaluate the influence of the external tension pattern, along with other parameters affecting myocardial mechanics, such as passive myocardial properties [26]. As the number of observed parameters increases, the parameter space becomes multidimensional and more challenging to analyze. Techniques such as manifold dimension reduction or similar methods could be employed to identify relationships within the parameter space [27–30]. However, these techniques are computationally intensive, and the computational time required may increase with the number of parameters. Therefore, fast computational models are essential.

Considering the unpredictability of myocardial pathology, it is crucial to simultaneously consider both AT and Con in extraction methods. Investigating the theoretical limitations of parameter extraction using computational models can provide valuable insights into the underlying system properties. The uniqueness and uncertainty of parameter extraction reflect the complex relationships between signals and underlying physiological parameters, presenting opportunities for improving extraction accuracy.

## 5. Conclusion

This study showed that the distribution of parameter values in the parameter space varies upon the position in the parameter space despite the initial inputs being always distributed stochastically. Furthermore, this distribution nonuniformity in the parameter space was studied and their features were utilized to improve parameter extraction. This was achieved by using a proximity map approach. The LBBB fingerprint is a conventional approach in parameter extraction and its characteristic is that it uses a stochastic concept of true value distribution, while the proximity map applies a nonuniform distribution, matching the complex system reality more accurately. The features of the distribution of nonuniformity in the parameter space provide new system insights and enhance myocardial tissue property extraction. This method has the potential to shift from intuition-based to quantitative, metrics-based clinical decision making.

## Figures and Tables

**Table 1:** Extracted tissue parameters using mean values from the data fingerprint and proximity map.

Measured strain	LBBB fingerprint		Proximity map	
	Time of activation (ms)	Contractility (%)	Time of activation (ms)	Contractility (%)
1	19 ± 15	73 ± 18	14 ± 44	95 ± 46
2	−19 ± 15	48 ± 7	−2 ± 45	85 ± 45
3	−27 ± 16	59 ± 7	−6 ± 44	89 ± 45
4	−6 ± 12	77 ± 13	2 ± 43	96 ± 45
5	−7 ± 8	110 ± 14	2 ± 43	110 ± 45
6	−59 ± 25	110 ± 18	−16 ± 46	109 ± 45

## Data Availability

All data used to support the findings of this study are available from the corresponding author upon request.
